# Facile Solvothermal Synthesis of CuCo_2_S_4_ Yolk-Shells and Their Visible-Light-Driven Photocatalytic Properties

**DOI:** 10.3390/ma11112303

**Published:** 2018-11-16

**Authors:** Yinxia Chen, Xianbing Ji, Vadivel Sethumathavan, Bappi Paul

**Affiliations:** 1 Hebei University of Environmental Engineering, Qinhuangdao 066102, China; chyxsd@163.com; 2 Department of Chemistry, PSG College of Technology, Coimbatore 641004, India; 3 Department of Chemistry, National Institute of Technology, Silchar, Assam 788010, India; bappipaulnits@gmail.com

**Keywords:** yolk-shell, structural, electron microscopy, semiconductors, photocatalyst

## Abstract

In this present work, we synthesized a yolk-shell shaped CuCo_2_S_4_ by a simple anion exchange method. The morphological and structural properties of the as-synthesized sample were characterized using X-ray diffraction (XRD), UV-vis diffuse reflectance spectra (UV-vis DRS), scanning electron microscopy (SEM), and transmission electron microscopy (TEM). The SEM and TEM results confirmed that the uniform yolk-shell structure was formed during the solvothermal process. The band gap was about 1.41 eV, which have been confirmed by UV–vis DRS analysis. The photocatalytic property was evaluated by the photocatalytic degradation of methylene blue (MB) dye as a target pollutant under the visible-light irradiation. The experimental results confirmed the potential application of yolk-shell shape CuCo_2_S_4_ in visible-light photocatalytic applications.

## 1. Introduction

Owing to their unique structural properties, as well as outstanding electrical properties, hollow nanostructures with tunable shape and size have gained huge research interest in the field of supercapacitors, catalysis, and biosensors [1]. The hollow architectures have relatively less density, high porosity, fast electron transfer, and higher surface area than the bulk materials. Up to date, various hollow structured metal oxides and sulfides have been successfully synthesized using soft and hard-template techniques [2]. For example, Y. Chen et al. fabricated Co_3_O_4_ hollow microspheres using Polyvinylpyrrolidone (PVP) as a template [3]. This synthesized hollow Co_3_O_4_ exhibits superior lithium storage capacity than the bulk Co_3_O_4_. S.E. Moosavifard et al. synthesized CuCo_2_O_4_ hollow spheres using a simple soft template route, which exhibits superior electrochemical than the bulk CuCo_2_O_4_ [4]. However, little attention has been paid by the research community to investigate the structural property of hollow metal sulfides [5]. In the past few decades, ternary metal sulfides have attracted great attention in the field of energy storage devices and photocatalysts due to their outstanding electrical properties. Among them, copper-cobalt sulfides (CuCo_2_S_4_) have been recognized as a promising material for pollutants degradation and water splitting, since they offer superior stability than other metal sulfides. CuCo_2_S_4_ is considered to be an environmentally benign material compared to CdS and In_2_S_3_ due to its low cost, less toxicity, and availability in nature [6]. In the past few decades, various morphologies of CuCo_2_S_4_, such as flowers, particles, and clusters have been successfully prepared using various approaches. In this work, we report a solvothermal route to synthesize a novel yolk-shell shaped CuCo_2_S_4_ using the sulfidation of Cu-Co glycerate precursor with thiourea. The prepared CuCo_2_S_4_ yolk-shell exhibited superior photocatalytic activity towards the degradation of MB dye under the visible light without the aid of co-catalysts. To the best of our knowledge, this is the first report on the synthesis of CuCo_2_S_4_ yolk-shells and applications in visible-light-driven photocatalysts.

## 2. Experimental Section

In a typical synthesis, 35 mg of Cu (NO_3_)_2_·3H_2_O, 72 mg of Co (NO_3_)_2_·6H_2_O was dissolved in a mixture of 7 mL of glycerol and 40 mL of isopropanol to form a clear solution. Then the solution was stirred for 1 h at room temperature and transferred into a Teflon-lined autoclave vessel and kept at 180 °C for 6 h in a muffle furnace. After cooling, the brown precipitate was washed thrice with ethanol and dried in a vacuum oven at 70 °C for 2 h and named as copper-cobalt glycerate precursor (Cu-Co glycerate). For the preparation of CuCo_2_S_4_ yolk-shell architectures, 30 mg of the Cu-Co-glycerate precursor was re-dispersed in 20 mL of ethanol solution, followed by the addition of 60 mg of thiourea. Then the mixture was transferred into a Teflon-lined autoclave and heated to 200 °C for 2 h. Finally, the black color product was washed with acetone several times, to obtain the CuCo_2_S_4_ (named as amorphous CuCo_2_S_4_). To improve the crystallinity, the final product was calcined at 300 °C for 30 min under air atmosphere (named as crystalline CuCo_2_S_4_).

### Materials Characterization

Crystallinity and phase purity were examined by X-ray powder diffraction (X’pert-PRO-PANalytical X-ray diffractometer operated at 40 Kv). The microstructures and morphologies of the CuCo_2_S_4_ were characterized using a scanning electron microscope (SEM: Zeiss EVO 18 electron microscope, Germany) and transmission electron microscopy (TEM: JEOL-JEM 2100, USA). Elemental mapping of the yolk-shell architectures was analyzed using the EDX attached to the TEM instrument. The UV-vis diffuse reflectance spectra (DRS) were analyzed using a JASCO spectrophotometer (V-750, Japan), using BaSO_4_ as a reference. The photocurrent measurements were recorded using a CHI660B workstation with a three-electrode configuration system. Then, 0.25 M Na_2_SO_4_ electrolytewas used for photocurrent measurements. The photocatalytic degradation of MB aqueous dye was carried out at room temperature under the visible-light irradiation of a 300 W tungsten halogen lamp. Then, 50 mg of catalyst and 50 mL of MB solution (10 ppm) was used for the photocatalytic degradation. At given intervals of illumination, the samples of the reaction solution were taken out, centrifuged, and analyzed using the JASCO UV-vis spectrophotometer.

## 3. Results and Discussion

**Figure 1:**
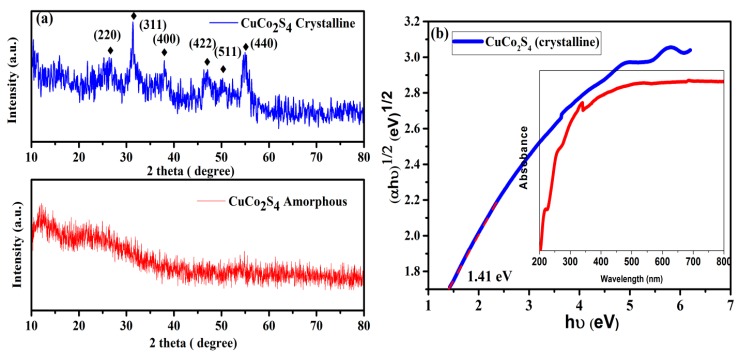
X-ray diffraction (XRD) patterns of (**a**) crystalline and amorphous CuCo_2_S_4_ (**b**) UV–vis diffuse reflectance spectra and (αhν)^2^ versus hν plots of crystalline CuCo_2_S_4_.

**Figure 2:**
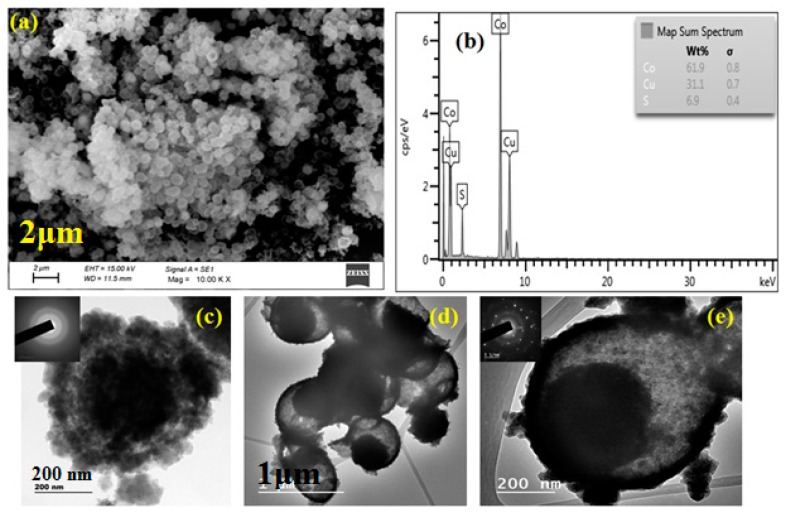
(**a**) Scanning electron microscopy (SEM) images (**b**) EDS spectra of CuCo_2_S_4_ yolk-shell structure (**c**) Transmission electron microscopy (TEM) images of amorphous CuCo_2_S_4_ (**d**,**e**) crystalline CuCo_2_S_4_ yolk-shell.

**Figure 3:**
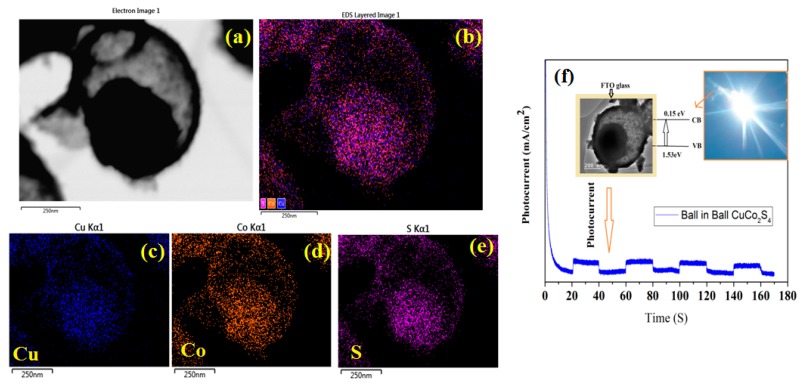
(**a**–**e**) EDS mapping (**f**) Photocurrent studies of CuCo_2_S_4_ yolk-shell structure.

The XRD pattern (Figure 1a) shows that all the diffraction peaks can be matched to the cubic phase of CuCo_2_S_4_ (crystalline) with JCPDS card No. 42-1450 [7]. Any other impurity peaks and secondary phases were observed, indicating that the Cu-Co-glycerate precursor was completely transferred to the CuCo_2_S_4_ phase after the sulfidation process with thiourea. According to the Scherrer calculation, the average crystalline size of CuCo_2_S_4_ was calculated to be 12 nm. The XRD pattern of amorphous CuCo_2_S_4_ (without calcination) did not show any prominent peaks indicating the poor crystalline nature of the material. Figure 1b shows the UV-vis DRS patterns of as-prepared CuCo_2_S_4_, which provides imperative evidence of their visible light photocatalytic performance. Apparently, the CuCo_2_S_4_ sample exhibited strong photo absorption in the entire visible light region. Using the Tauc plot of (αhν)^1/2^ vs. photon energy (hν), the band gap value for the CuCo_2_S_4_ yolk-shell was calculated to be 1.41 eV, respectively. SEM analysis was employed to investigate the morphology and the detailed structural property of the CuCo_2_S_4_ yolk-shells. SEM images from Figure 2a indicating a well-preserved spherical morphology with average diameters of about 1µm were observed. The interior structure of the yolk-shell structure was further investigated by TEM analysis. As shown in Figure 2d,e, the TEM images revealed that the CuCo_2_S_4_ composed of many hollow spherical shells with a relatively darker core could be observed in each of the spheres (Figure 2c). Moreover, the amorphous CuCo_2_S_4_ did not show any uniform yolk-shell formation. Furthermore, the selected area electron diffraction (SAED) pattern (the inset of Figure 2e) determined that the yolk-shell CuCo_2_S_4_ was existing in polycrystalline nature. The TEM observation clearly confirmed that the as-prepared CuCo_2_S_4_ yolk-shells consisted of a highly porous nature which may be beneficial for superior photocatalytic degradation. Furthermore, the EDS (Figure 3a–e) images clearly indicate that the Cu, Co, and S elements are uniformly distributed in the CuCo_2_S_4_ yolk-shells, as in Reference [8]. These unique yolk-shell structures possessed a porous structure with a large specific surface area, guaranteeing a large contact area between the dye and the photocatalyst. Moreover, the electron separation and transporting behavior of the excited electron-hole pairs in CuCo_2_S_4_ could be validated by photocurrent studies. An appreciable photocurrent value can generally originate from the enhanced charge transportation and separation efficiency of photo-generated carriers [9]. As outlined in Figure 3f, the CuCo_2_S_4_ yolk-shells show an obvious photocurrent value, which implies that the effective charge separation in the CuCo_2_S_4_ system may beneficial for superior photocatalytic activity.

**Figure 4:**
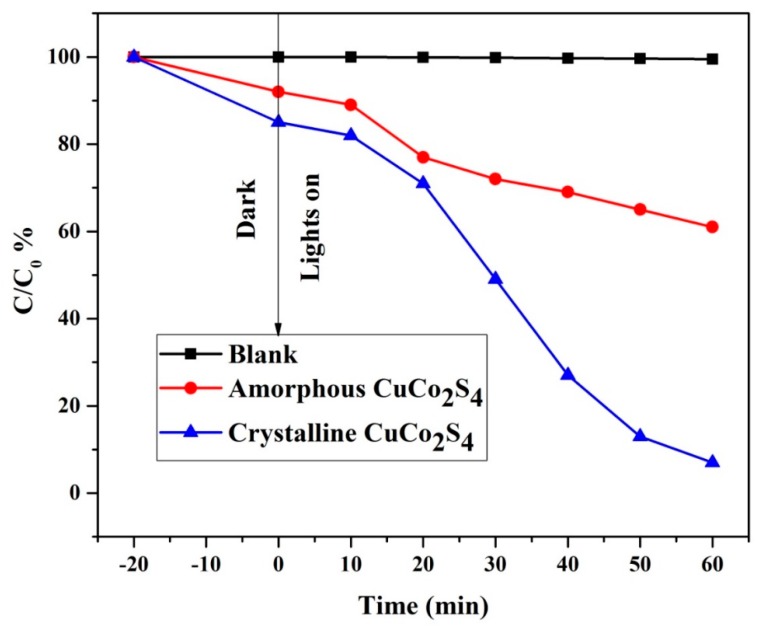
Photodegradation of MB with amorphous and crystalline CuCo_2_S_4_ under visible-light irradiation.

Photocatalytic properties of the CuCo_2_S_4_ sample were investigated using the degradation of methylene blue (MB) dye as a target pollutant, Figure 4 shows the photocatalytic degradation curves (C_t_/C_0_) in % of MB aqueous dye solution with crystalline and amorphous CuCo_2_S_4_ as visible-light photocatalyst. For the blank test without the aid of any photocatalysts, the concentration of MB dye was not changed during the entire period of visible-light irradiation. After adsorption-desorption attainment in the dark, the change in the concentration of MB dye was only about 16% for crystalline CuCo_2_S_4_ material which was higher than that of amorphous CuCo_2_S_4_ powder (about 8%). However, the visible-light degradation of the crystalline CuCo_2_S_4_ was 95% after the 45 min visible-light irradiation, whilst that for the amorphous CuCo_2_S_4_ photocatalyst degraded only 38% after 45 min light irradiation [10,11,12]. Hence, crystalline CuCo_2_S_4_ had a higher photocatalytic activity than amorphous CuCo_2_S_4_ towards the MB degradation due to its pronounced oxygen vacancy and higher surface area (Figures S1 and S2). 

**Figure 5:**
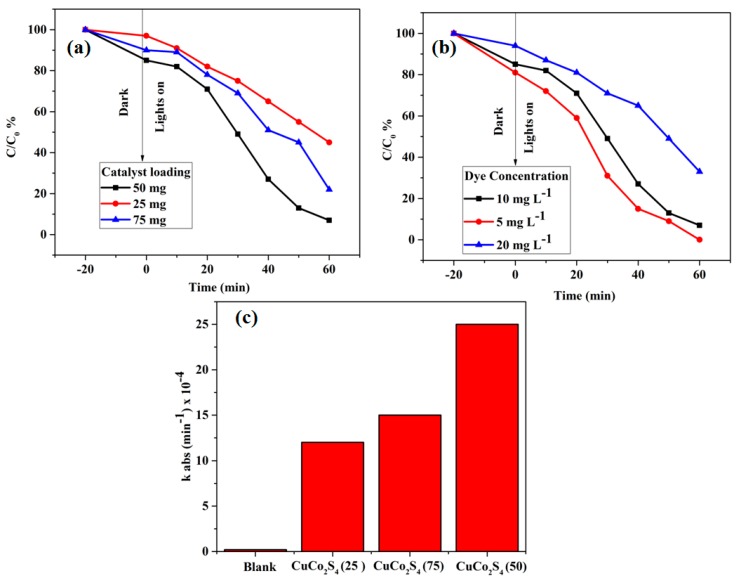
(**a**) Photodegradation of MB dye under different amounts of crystalline CuCo_2_S_4_ (**b**) different concentration of MB dye on CuCo_2_S_4_ (50 mg). (**c**) Degradation rate constant of MB over the CuCo_2_S_4_ yolk-shells loading.

**Figure 6:**
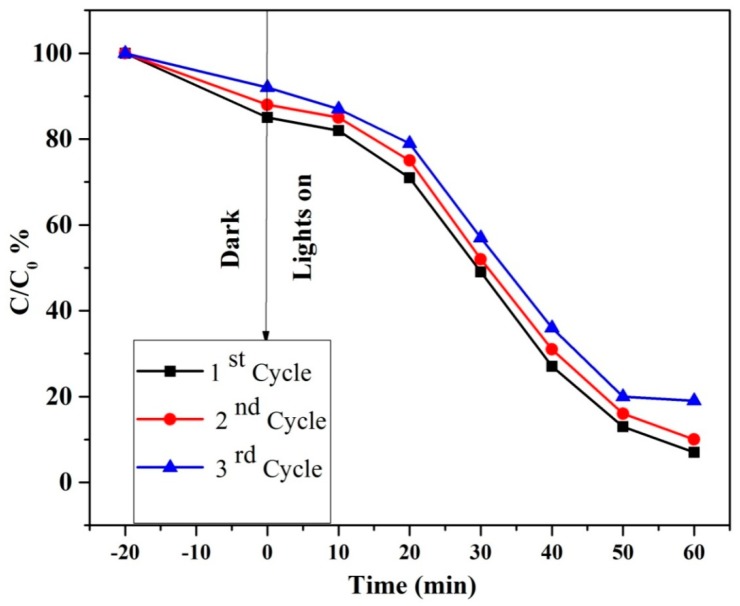
Cycle runs of MB photocatalytic degradation in the presence of crystalline CuCo_2_S_4_.

As shown in Figure 5a, CuCo_2_S_4_ was evaluated by varying the photocatalyst loadings from 25 to 75 mg at 10 mg L^−1^ of MB concentration. Initially, the degradation efficiency was increased with an increased number of catalytic loadings, which may be due to the availability of active sites for electron-hole pair generation. Beyond the 50 mg of photocatalyst loadings, the degradation efficiency was comparatively lower than the 50 mg loadings, which may be due to reduced light penetration in the photocatalyst system. The effect of dye concentration on the photocatalytic properties of CuCo_2_S_4_ was studied by varying the concentrations from 5 mg L^−1^ to 20 mg L^−1^, as shown in Figure 5b. It was observed that 5 mg L^−1^ of MB dye was completely degraded within 60 min. Beyond 10 mg L^−1^, the degradation efficiency of CuCo_2_S_4_ was much lower, which may be due to higher concentration of dye molecules blocking light penetration in the photocatalytic system. Figure 5c shows that the degradation rate constant of MB over the mg loading of CuCo_2_S_4_ (25), CuCo_2_S_4_ (50), and CuCo_2_S_4_ (75), was 12, 15, and 25 min^−1^ × 10^−4^, respectively. Hence, the proper loading amount of CuCo_2_S_4_ (50%) photocatalytic showed higher photocatalytic activity than other loadings. In addition, as shown in Figure 6, the cycle test results illustrate that the crystalline CuCo_2_S_4_ had a lower loss in its activity after three cycles [13,14,15,16,17,18,19,20]. Therefore, crystalline CuCo_2_S_4_ yolk-shells powder would be an ideal candidate for visible-light photocatalytic applications. Some few examples of copper based photocatalyst materials and its comparison with the CuCo_2_S_4_ yolk-shells are shown in Table 1.

## 4. Conclusions 

In summary, a simple solvothermal route was implemented for the preparation of CuCo_2_S_4_ yolk-shell structures. The UV-DRS spectra showed that the CuCo_2_S_4_ architectures had a broad visible-light absorption spectrum. The prepared CuCo_2_S_4_ yolk-shell structure exhibited good visible-light response and excellent photocatalytic activity towards the degradation of MB dye. The photocatalytic performances of the CuCo_2_S_4_ architectures were better than the previously reported degradation rate, which infers the potential applications of CuCo_2_S_4_. These results provided new insights that might lead to the development of CuCo_2_S_4_ yolk-shells for further application in the field of environmental remediation and energy storage devices, to utilize solar energy effectively.

## Figures and Tables

**Table 1:** Summary of photocatalytic performance of copper based photocatalyst materials.

S.No	Compound	Synthesis Method	Light Source	Dye	Conc mg L^−1^	Catalyst Dosage	Irradiation Time	Efficiency	Ref.
1	Cu_2_SnS_3_	Solvothermal	100 W Xe Lamp	Methylene Blue	5	20 mg	240 min	N/A	[21]
2	Cu_2_SnS_3_	Solvothermal	150 W Tungsten–Halogen Lamp	Eosin	7.5	100 mg	140 min	N/A	[22]
3	Cu_2_SnS_3_	Hydrothermal	500 W Xe Lamp	Rhodamine B	10	100 mg	210 min	50%	[23]
4	CuCo_2_S_4_	Hydrothermal	500 W Tungsten–Halogen Lamp	Malachite Green	10	50 mg	360 min	40%	[24]
5	CoS_2_	Hydrothermal	8 W Halogen Lamp	Methylene Blue	5	50 mg	120 min	N/A	[25]
6	Hexagonal Cu_2_S copper sheets	N/A	300 W Xe Lamp	Rhodamine B	10	N/A	60 min	96%	[26]
7	Yolk-shell CuCo_2_S_4_	Ion exchange route	300 W Tungsten Halogen Lamp	Methylene Blue	10	50 mg	60 min	95%	This work

## Supplementary Materials

The following are available online at http://www.mdpi.com/1996-1944/11/11/2303/s1, Figure S1: BET spectrum of CuCo_2_S_4_ yolk-shells; Figure S2: XPS spectrum of CuCo_2_S_4_ yolk-shells; Figure S3: FT-IR spectrum of CuCo_2_S_4_ yolk-shells; Figure S4: SEM, TEM and EDX of amorphous CuCo_2_S_4_.
