# Supplementary material for: Facile Solvothermal Synthesis of CuCo2S4 Yolk-Shells and Their Visible-Light-Driven Photocatalytic Properties
Source: Materials (Basel). 2018 Nov 16;11(11):2303. doi: 10.3390/ma11112303 (PMC6265846; doi:10.3390/ma11112303)
Supplement: Supplementary file 1 [file materials-11-02303-s001.pdf]

materials

MDPI

Facile solvothermal synthesis of $CuCo_2S_4$ yolk-shells and their visible-light-driven photocatalytic properties

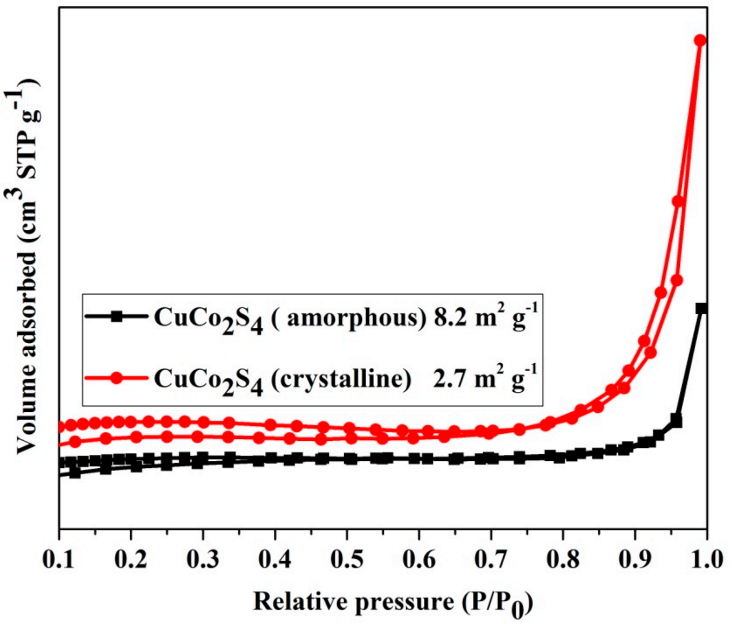

Fig.S1 BET spectrum of $CuCo_2S_4$ yolk-shells

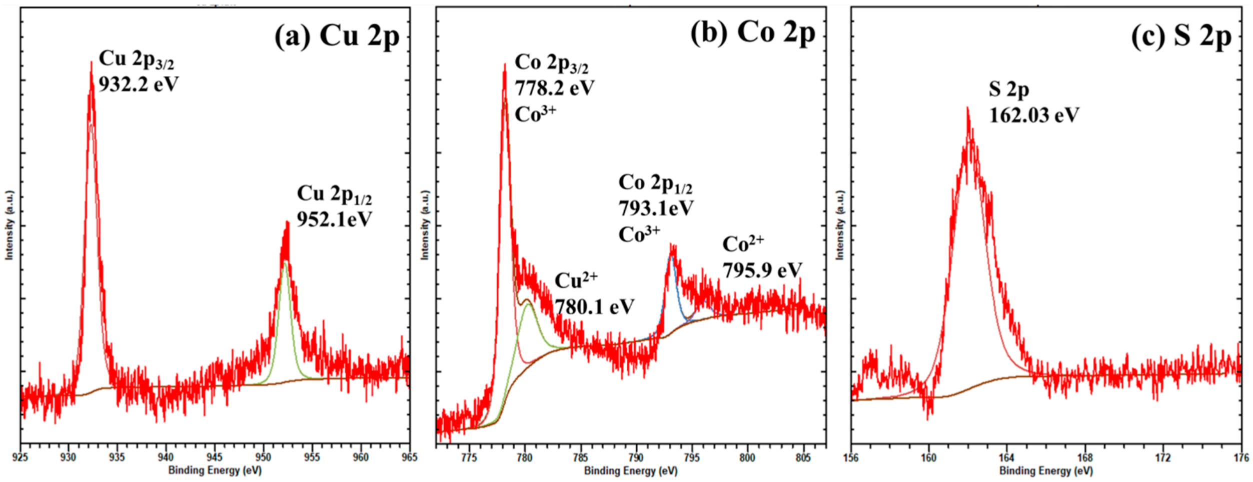

Fig: S2 XPS spectrum of $CuCo_2S_4$ yolk-shells

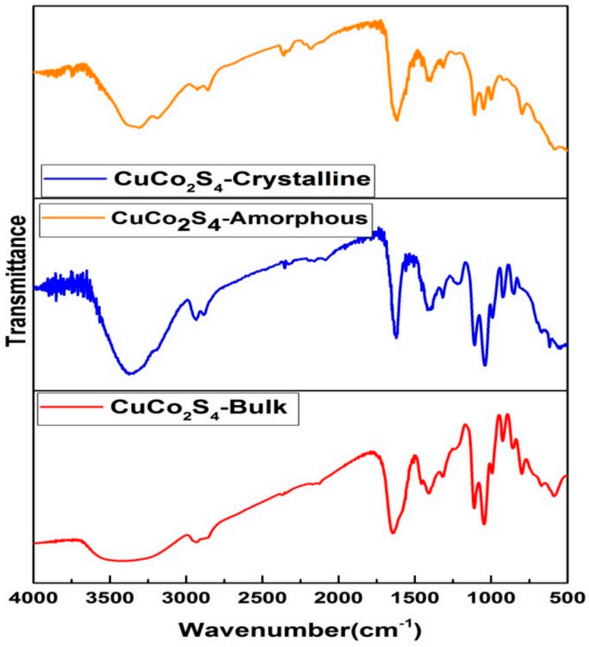

Fig: S3 FT-IR spectrum of $CuCo_2S_4$ yolk-shells

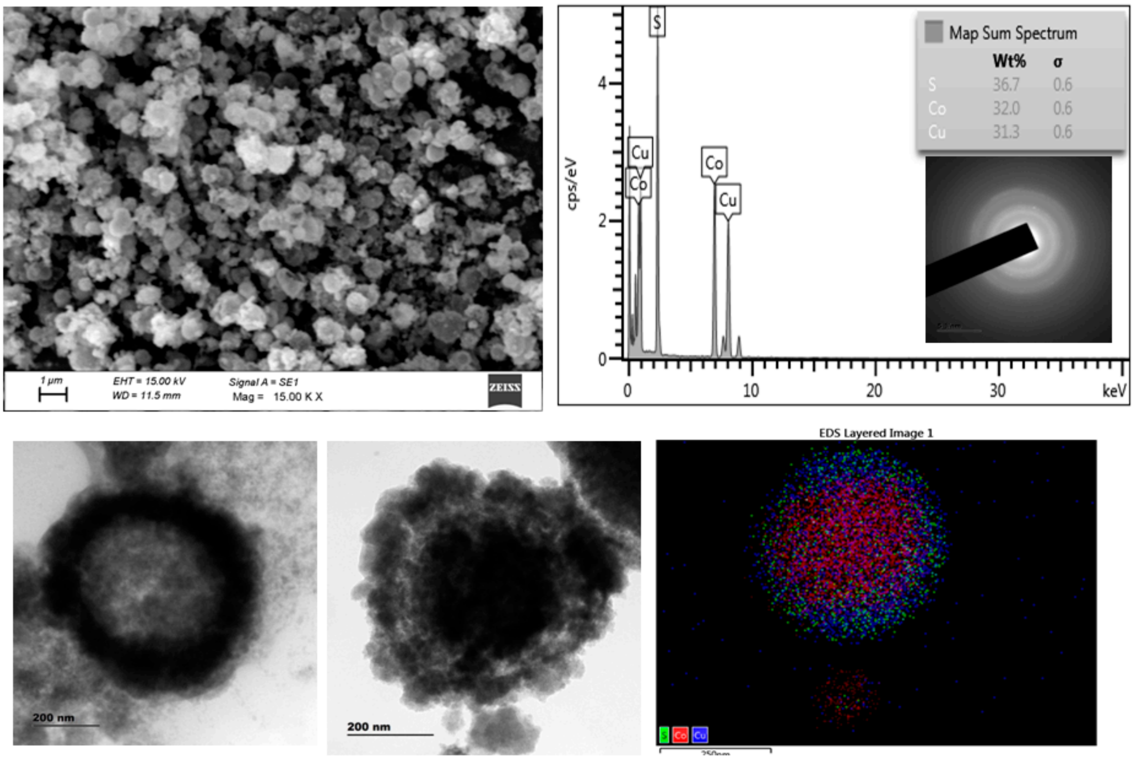

Fig: S4, SEM, TEM and EDX of amorphous $CuCo_2S_4$
